# Micronucleus frequency among Iraqi thyroid disorder patients

**DOI:** 10.1007/s00580-012-1671-7

**Published:** 2012-12-28

**Authors:** Abdul Hussein Moyet AlFaisal, Intesar Jawad Kahdoom AL-Ramahi, Ismail Abdul Redah Abdul-Hassan

**Affiliations:** 1 Genetic Engineering and Biotechnology Institute, Baghdad, Iraq; 2 Al-Razi Centre for Medical Diagnostic kits Production, Ministry of Industry, Baghdad, Iraq

**Keywords:** Thyroid disorders, Micronucleus, NDI, BN

## Abstract

Micronucleus (MN) assay has been extensively used in detection of DNA damage, instability in cancer, and genetic disorders. In the current study, MN, binucleated cells, and nuclear division index (NDI) were investigated in Iraqi patients with thyroid disorders. The results indicated significantly (*p* < 0.05) increased binucleated cells with micronucleus (BNMN) frequencies in thyroid cancer group (37.58 ± 3.07) versus other thyroid disorder groups (6.60 ± 1.29, 14.90 ± 1.69, 15.56 ± 1.76). On the other hand, the frequency of micronucleus per 1,000 and the NDI were significantly (*p* < 0.05) decreased in hypothyroidism (MN 1.55 ± 0.36) (NDI 0.009 ± 0.001) versus other thyroid disorder groups (MN: 6.05 ± 0.97, 6.09 ± 0.53, 5.34 ± 0.56) (NDI: 0.049 ± 0.003, 0.032 ± 0.002, 0.025 ± 0.002), with no difference versus healthy group (0.0 ± 0.0). The number of BNMN and MN are parallel to the severity of thyroid disorders which were 6.60 ± 1.29, 14.90 ± 1.69, 15.56 ± 1.76, and 37.58 ± 3.07 for hypothyroidism, thyroid toxic goiter, thyroid nontoxic goiter, and thyroid cancer, respectively. The number of BNMN and MN are parallel to the severity of thyroid disorders which were 6.60 ± 1.29, 14.90 ± 1.69, 15.56 ± 1.76, and 37.58 ± 3.07 for hypothyroidism, thyroid toxic goiter, thyroid nontoxic goiter, and thyroid cancer, respectively. The results also indicate that there were no significant differences among age and sex groups as related with BNMN formation within each thyroid disorder groups and healthy control group.

## Introduction

Chromosomal damages measurement is one of important ways to evaluate the toxicity, carcinogenicity, and mutagenicity of drugs, chemicals, and rays (Cotterill et al. 2001; Ge et al. 2005; Gad and Saad 2008). Such damages were also detected to associate with some diseases and cancer (Fenech 2000; Neri et al. 2005; El-Zein et al. 2008, 2011). Most of these damages appeared as gene mutations and chromosomal rearrangements or appeared as free genetic particles near nucleus such as micronucleus or genetic balls distributed along cell cytoplasm such as double minutes (Gil et al. 2000; Herrmann 2003; Joseph et al. 2009). Many protocols were used to evaluate the genetic effects of these harmful factors; most of them are long, complicated, and costly assays. Micronucleus assay proofed to be the preferred method for assessing chromosome damage because they enable both chromosome loss and chromosome breakage to be measured reliably (Fenech 2000; 2002; Joseph et al. 2009). Micronucleus (MN) is arise during cell division when a whole lagging acentric chromosome or chromosome with nonfunctional centromere or damaged chromosomes (Leach and Cook 2004; AlFaisal 2007; AlFaisal et al. 2010). These oval or circular bodies do not integrate to the daughter nuclei and appeared as highly stained bodies beside nucleus membrane. The principle of the micronucleus assay is by adding of cytochalasin B to the cell cultures to block cytokinesis cell cultures which lead to the formation of micronuclei in bi- or multinucleated interphase cells (Fenech 2000). This assay has been extensively used in routine mutagen and carcinogen screening protocols to detect factors and agents that cause chromosomal damages and cytological toxicity as well to evaluate the genetical and cytological damages to lymphocytes of cancer patients before and after radiotherapy (Hooman et al. 2008). Recently, COMET assay was applied to detect DNA damages in thyroid patients before and after treatment (Ge et al. 2005).

The present study was performed to determine the genetic damages and DNA instability via MN assay associated with various types of thyroid disorders.

## Material and methods

### Subjects

Five study groups have been investigated. Apparently healthy control group consists of 25 healthy individuals of different ages. No obvious abnormalities were selected from blood bank donors for comparison. One hundred patients with thyroid disorders who attend the endocrinologist in Nuclear Medicine Hospital and Al Yarmok Nuclear Medicine Department in Baghdad, Iraq were selected. Clinical, ultrasonication, and serum thyroid hormones were used for diagnosis. Healthy controls and patients’ ages ranged from 17 to 79 years. All patients were suffering from thyroid disorders such as toxic goiter, thyroid nontoxic goiter, thyroid hypothyroidism, and thyroid cancer in Baghdad during a period from July 2009 to October 2009.

### Blood samples

Venous blood sample (3 ml) was collected in heparinized tubes by trained nurses from each individual of both thyroid disorder and healthy control groups.

### Blood culture protocol

Blood culture protocol was done according to Fenech (2000). A 0.5-ml blood sample was added to culture tubes containing 4.5 ml of RPMI 1640 media enriched with 20 % fetal calf serum and 0.2 ml of phytohemagglutinin 1 % in each. Tubes were mixed gently by inverting a few minutes and incubated for 44 h at 37 °C in a slant position. Cytochalasin B was then added to each culture at a concentration of 3 μg/ml to block cell cytokinesis, and cultures were reincubated at 37 °C for further 28 h. Cells were then harvested by centrifugation at 2,000 rpm for 10 min. Supernatant was discarded by pipetting the media, leaving a little medium as possible over the cell pellet. Cell pellet was resuspended in the supernatant remains, and 10 ml of warm hypotonic (0.075 M KCl) solution was added gently to each tube. Tubes were then mixed and incubated for 30 min in a water bath at 37 °C. The tubes were centrifuged at 2,000 rpm for 10 min; the supernatant was discarded; and the pellet was resuspended in the supernatant remains, and 5 ml of fixative solution was added gently to each tube. Tubes were kept in refrigerator for about 30 min. The tubes were centrifuged, and the supernatant was discarded. Fixation steps were repeated for three more times. After the final centrifugation, the cells were resuspended in a small volume of fixative solution (approximately 0.5–1 ml) depending on the size of cell bottom to give a slightly opaque suspension.

### Slide preparation and estimation

Slides were prepared in order to examine the micronuclei formation and the nuclear division index. Before use, the slides were cleaned well with methanol then with distilled water. After that, the fixed lymphocyte cells were dropped from about 30-cm height using a Pasteur pipette onto slides which were dried at 37 °C (Lamberti et al. 1983), stained with Giemsa stain, and examined by light microscope (×40 and × 100). At least 1,000 binucleated cells per duplicate cell culture were scored to assess the frequency of cells with one, two, or more than two micronuclei. Additionally, the cells were classified as mononucleates, binucleates, or multinucleates (Kirsch-Volders et al. 1997). The frequency of binucleate and micronuclei were calculated as follow:

$$ \begin{array}{*{20}c} {{{{\mathrm{Binucleated}\;\mathrm{cells}\;\mathrm{with}\;\mathrm{micronucleus}\;\left( {\mathrm{BNMN}} \right)\ }} \left/ {{1,000\;\mathrm{binucleated}\;\mathrm{cells}\;\left( {1,000\;\mathrm{BN}} \right)}} \right.}} \hfill \\ {{{{\mathrm{Number}\;\mathrm{of}\;\mathrm{MN}}} \left/ {{1,000\;\mathrm{binucleated}\;\mathrm{cells}\;\left( {1,000\;\mathrm{BN}} \right)}} \right.}} \hfill \\ \end{array} $$

### Nuclear division index

The proliferation index was estimated by measuring the nuclear division index according to Lamberti et al. (1983).

$$ \begin{array}{*{20}c} {\mathrm{NDI}={{{\left[ {1\left( {\mathrm{M}1\%} \right)+2\left( {\mathrm{M}2\%} \right)+3\left( {\mathrm{M}3\%} \right)+4\left( {\mathrm{M}4\%} \right)} \right]}} \left/ {N} \right.}} \hfill \\ {\mathrm{M}\mathrm{N}=\left[ {{{{1\left( {\mathrm{M}\mathrm{N}1} \right)+2\left( {\mathrm{M}\mathrm{N}2} \right)+3\left( {\mathrm{M}\mathrm{N}3} \right)+4\left( {\mathrm{M}\mathrm{N}4} \right)}} \left/ {N} \right.}} \right]} \hfill \\ \end{array} $$

NDI: Nuclear division index.
M, 1, 2, 3, 4: Number of binucleated cells with micronucleus.
MN, 1, 2, 3, 4: Number of micronucleus in binucleated cells.
*N*: Total number of cells.

## Results

**Fig. 1:**
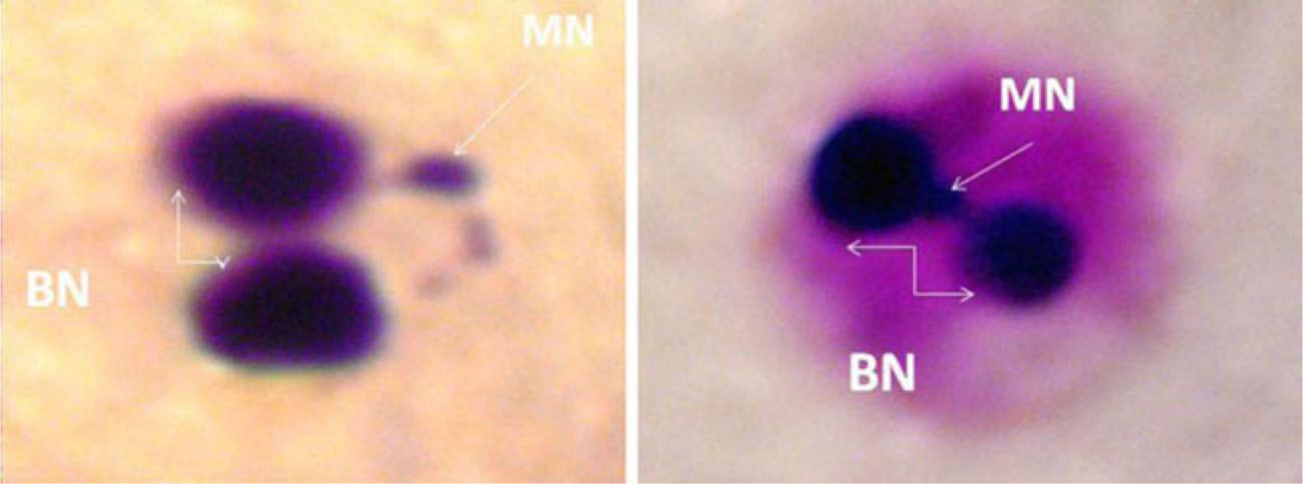
Binucleate (BN) cells with Micronucleus (*MN*) in thyroid disorders patients (×100)

A total number of BNMN per 1,000 and MN per 1,000 were calculated for each patient group and compared with healthy (control) group (Fig. 1).

The numbers of micronuclei per 1,000, binucleated cell with micronuclei per 1,000, and NDI in peripheral blood lymphocytes of Iraqi thyroid disorders compared with healthy control group are summarized in Table 1.

**Table 1:** Frequency of BNMN, MN, and NDI (mean ± SE) among Iraqi thyroid disorders patients and apparently healthy control

Groups	NDI	Micronucleus MN/1,000	BNMN/1,000
Hypothyroidism	0.009 ± 0.001 b	1.55 ± 0.36 b	6.60 ± 1.29 c
Thyroid Cancer	0.049 ± 0.003 a	6.05 ± 0.97 a	37.58 ± 3.07a
Thyroid Toxic goiter	0.032 ± 0.002 a	6.09 ± 0.53 a	14.90 ± 1.69 b
Thyroid Nontoxic goiter	0.025 ± 0.002 a	5.34 ± 0.56 a	15.56 ± 1.76 b
Healthy control	0.000 ± 0.000 c	0.0 ± 0.0 b	0.000 ± 0.000 d

Significant differences at (*p* < 0.05). Different letters refer to significant differences among means

*BNMN* Binucleate cells with micronucleus, *MN* Micronucleus, *NDI* Nuclear Division Index

The results indicated significantly (*p* < 0.05) increased BNMN frequencies in thyroid cancer group (37.58 ± 3.07) versus hypothyroidism, thyroid toxic goiter, and thyroid nontoxic goiter groups (6.60 ± 1.29, 14.90 ± 1.69, and 15.56 ± 1.76, respectively). Also, significant (*p* < 0.05) increase in other thyroid disorder groups versus healthy control (0.0 ± 0.0) were observed. While the frequency of micronucleus per 1,000 significantly (*p* < 0.05) decreased in hypothyroidism (1.55 ± 0.36) versus other thyroid disorder groups (6.05 ± 0.97, 6.09 ± 0.53, 5.34 ± 0.56), with no difference in the frequency of micronucleus per 1,000 between hypothyroidism group and healthy group (0.0 ± 0.0). In addition, there were no significant differences in micronuclei per 1,000 among thyroid cancer, thyroid toxic goiter, and nontoxic goiter (6.05 ± 0.97, 6.09 ± 0.53, and 5.34 ± 0.56 respectively).

The NDI frequencies were significantly decreased in hypothyroidism (0.009 ± 0.001) compared with thyroid cancer, thyroid toxic, and nontoxic goiter groups (0.049 ± 0.003, 0.032 ± 0.002, and 0.025 ± 0.002, respectively). There is no significant difference in NDI frequencies among thyroid cancer, thyroid toxic, and thyroid nontoxic goiter (0.049 ± 0.003, 0.032 ± 0.002, and 0.025 ± 0.002, respectively).

The number of BNMN and MN are parallel to the severity of thyroid disorders which were 6.60 ± 1.29, 14.90 ± 1.69, 15.56 ± 1.76, and 37.58 ± 3.07 for hypothyroidism, thyroid toxic goiter, thyroid nontoxic goiter, and thyroid cancer, respectively.

Tables 2 and 3 illustrated results of related micronuclei formation rates with age and sex groups. There were no significant differences among age and sex groups as related with BNMN formation within each thyroid disorder groups and healthy control group (Table 2).

**Table 2:** Frequencies of BNMN, MN, and NDI (mean ± SE) among Iraqi thyroid disorders patients according to age

Group\Parameter	Age year			
	<30	30–50	>50	LSD (ns)
Hypothyroidism				
BNMN/1,000	1.00 ± 0.18	1.50 ± 0.32	2.16 ± 0.83	2.003
MN/1,000	7.00 ± 2.86	6.50 ± 1.50	6.33 ± 2.95	7.370
NDI	0.009 ± 0.002	0.009 ± 0.001	0.01 ± 0.004	0.009
Thyroid cancer				
BNMN/1,000	7.00 ± 2.79	5.72 ± 1.20	6.00 ± 1.00	7.160
MN/1,000	37.50 ± 4.33	39.27 ± 4.14	28.50 ± 11.50	20.541
NDI	0.042 ± 0.002	0.051 ± 0.004	0.052 ± 0.002	0.022
Thyroid toxic goiter				
BNMN/1,000	6.25 ± 0.81	6.68 ± 0.82	4.57 ± 0.97	2.738
MN/1,000	12.12 ± 3.58	14.31 ± 1.76	19.42 ± 4.85	9.032
NDI	0.034 ± 0.004	0.0034 ± 0.003	0.026 ± 0.004	0.011
Thyroid nontoxic goiter				
BNMN/1,000	3.50 ± 1.50	4.94 ± 0.73	6.15 ± 0.95	4.249
MN/1,000	19.50 ± 13.50	15.94 ± 2.88	14.46 ± 1.64	12.812
NDI	0.026 ± 0.016	0.024 ± 0.003	0.026 ± 0.003	0.019

*Ns* nonsignificant

**Table 3:** Frequencies of BNMN, MN, and NDI (mean ± SE) between males and females of Iraqi thyroid disorders patients

Group\Parameter	Males	Females	LSD (ns)
Hypothyroidism			
BNMN/1,000	1.66 ± 0.95	1.50 ± 0.35	1.767
MN/1,000	6.83 ± 3.09	6.50 ± 1.38	6.506
NDI	0.009 ± 0.001	0.010 ± 0.004	0.008
Thyroid Cancer			
BNMN/1,000	6.83 ± 1.81	5.63 ± 1.19	4.853
MN/1,000	41.66 ± 3.80	35.36 ± 4.24	13.923
NDI	0.032 ± 0.002	0.032 ± 0.003	0.009
Thyroid Toxic Goiter			
BNMN/1,000	5.16 ± 0.42	6.68 ± 0.80	2.151
MN/1,000	12.58 ± 1.79	16.36 ± 2.49	7.096
NDI	0.032 ± 0.003	0.0.032 ± 0.002	0.009
Thyroid Nontoxic Goiter			
BNMN/1,000	3.50 ± 1.19	5.60 ± 0.61	3.488
MN/1,000	6.50 ± 2.53	16.85 ± 1.87	10.519
NDI	0.03 ± 0.002	0.013 ± 0.005	0.015

*Ns* nonsignificant, *BNMN* Binucleated cells with micronucleus, *MN* Micronucleus, *NDI* Nucleic Division Index

## Discussion

MN frequency is a biomarker of chromosomal damage, genome instability, and cancer risk that integrates acquired mutations and genetic susceptibility (Fenech 2000; Joseph et al. 2009). In addition, the NDI and the proportion of binucleated cells are biomarkers of mutagen response and immune function in lymphocytes as well as cytostatic effects of agents (Kocaman et al. 2008; Bonassi et al. 2011; El-Zein et al. 2011). In thyroid disorders, micronucleus assay was used as well to other cytogenetic assays such as chromosomal aberrations assay (Wilkens et al. 2000; Hooman et al. 2008), micronuclei assays associated with fluorescence in situ hybridization (FISH) ( Joseph et al. 2009), and COMET assay (Ge et al. 2005).

The current results indicated that significantly increased BNMN frequencies in thyroid cancer group versus other thyroid disorder groups which reflect a high proliferative rate, high level of genetic damage and cytotoxicity of cancer, and less rate but significant in other groups. The results also showed that a significant number of micronuclei and NDI was detected in all thyroid disorder groups versus healthy. The NDI was significantly higher in cancer, toxic goiter, and nontoxic goiter than hypothyroidism and healthy control. These results indicated a high DNA instability and DNA damages associated with thyroid disorders especially in thyroid cancer and toxic goiter which may reflect the possibility of chronic exposure to genotoxic agents such as radiations. Such genotoxicity was also detected as mutations in TPO and TG genes among Iraqi thyroid disorders patients (AlFaisal et al. 2012; Al-Ramahi et al. 2012). After Gulf war I in 1981, Gulf war II in 1991, and Gulf war III in 2003, the Iraqi environment radiation contamination was well documented (Al-Azzawi and Al-Saji 1999; IFAM 1995; Al-Azzawi et al. 1999, 2002; Butrus et al. 2002), and the incidences of various types of cancer and genetic disorders due to this contamination were arisen (Al-Sadoon et al. 1998; Yaqoub et al. 1998a, b, 1999, 2002; Ali and Al-Ali 2002). This possibility is supported by the genotoxicity associated with radiations which were observed by several studies. Such studies have investigated the level of genome damage and micronucleus in thyroid cancer patients environmentally exposed to radiation after the Chernobyl fallout (Sbrana et al. 2006; Tronko et al. 2007; Cardis and Hatch 2011), after Fukushema in Japan (Medalia 2011), in patients affected by thyroid cancer who underwent radiotherapy (Livingston et al. 1993; Gutierrez et al. 1997, 1999; Dardano et al. 2007; Hooman et al. 2008), and in people affected by thyroid nodules following occupational exposure to ionizing radiation (Brooks et al. 2007; Scarpato et al. 2009).

The results of the current study also showed that no significant differences among age and sex groups as related with BNMN formation within each thyroid disorder groups and healthy control group. These results are correspondents to those by Scarpato et al. (2011) who reported that gender and age did not cause variation in the MN baseline level. Other literature regarding gender and MN showed that the frequencies of MN are greater in females than in males (Thierens et al. 2000; Joseph et al. 2009).
